# Dysregulated autophagy contributes to caspase-dependent neuronal apoptosis

**DOI:** 10.1038/s41419-018-1229-y

**Published:** 2018-12-11

**Authors:** Yuhyun Chung, Juhyung Lee, Shinae Jung, Yangsin Lee, Jin Won Cho, Young J. Oh

**Affiliations:** 1 Department of Systems Biology, Yonsei University College of Life Science and Biotechnology, Seoul, 120-749 South Korea; 2 Glycosylation Network Research Center, Yonsei University, Seoul, 120-749 South Korea; 3 Interdisciplinary Program of Integrated OMICS for Biomedical Science, Yonsei University, Seoul, 120-749 South Korea

## Abstract

Autophagy is a regulated, intracellular degradation process that delivers unnecessary or dysfunctional cargo to the lysosome. Autophagy has been viewed as an adaptive survival response to various stresses, whereas in other cases, it promotes cell death. Therefore, both deficient and excessive autophagy may lead to cell death. In this study, we specifically attempted to explore whether and how dysregulated autophagy contributes to caspase-dependent neuronal cell death induced by the neurotoxin 6-hydroxydopamine (6-OHDA). Ultrastructural and biochemical analyses indicated that MN9D neuronal cells and primary cultures of cortical neurons challenged with 6-OHDA displayed typical features of autophagy. Cotreatment with chloroquine and monitoring autophagic flux by a tandem mRFP-EGFP-tagged LC3 probe indicated that the autophagic phenomena were primarily caused by dysregulated autophagic flux. Consequently, cotreatment with an antioxidant but not with a pan-caspase inhibitor significantly blocked 6-OHDA-stimulated dysregulated autophagy. These results indicated that 6-OHDA-induced generation of reactive oxygen species (ROS) played a critical role in triggering neuronal death by causing dysregulated autophagy and subsequent caspase-dependent apoptosis. The results of the MTT reduction, caspase-3 activation, and TUNEL assays indicated that pharmacological inhibition of autophagy using 3-methyladenine or deletion of the autophagy-related gene *Atg5* significantly inhibited 6-OHDA-induced cell death. Taken together, our results suggest that abnormal induction of autophagic flux promotes apoptotic neuronal cell death, and that the treatments limiting dysregulated autophagy may have a strong neuroprotective potential.

## Introduction

Autophagy is a highly conserved cellular degradative process that involves the delivery of cytoplasmic substrates to the lysosomes^1^. There are three types of autophagy: macroautophagy, chaperone-mediated autophagy, and microautophagy. In macroautophagy, the targeted cytoplasmic constituents are wrapped around by the intermediary double-membrane bound vesicle called autophagosome. The autophagosome fuses with the lysosome for degradation or recycling cytoplasmic cargos. It has been recently shown that autophagy plays a wide variety of physiological and pathophysiological roles in mammalian cells^2,3^. Therefore, physiological levels of autophagy must be tightly regulated because both impaired and excessive autophagy promotes cell death^4–6^. It has been demonstrated that autophagy plays an important role in various neurodegenerative disorders, such as Parkinson’s disease (PD), Alzheimer’s disease, and Huntington’s disease^7–9^. Whether autophagy has cytoprotective^10–12^ or cytotoxic^13,14^ effects in neurodegenerative diseases remains controversial. Intriguingly, it has been proposed that the interplay between autophagy and apoptosis may contribute to neurodegeneration^15–17^.

Neurotoxin-based experimental models have been used to study biochemical changes reminiscent of those occurring in patients with PD^18^. Among such neurotoxins, 6-hydroxydopamine (6-OHDA) has been first introduced^19^. 6-OHDA is structurally similar to dopamine; it can penetrate monoaminergic neurons via dopamine and norepinephrine transporters and cause their death^20^. It has been indicated that 6-OHDA-induced toxicity is primarily ascribed to the oxidative stress generated by reactive oxygen species (ROS) and subsequent inactivation of biological macromolecules^21^. Numerous studies have demonstrated that 6-OHDA-treated neurons undergo apoptotic cell death^22–24^, whereas others have indicated that 6-OHDA treatment also induces autophagy in dopaminergic neurons^13,25^. Previously, we demonstrated that ROS-triggered apoptotic signaling is responsible for 6-OHDA-induced neurodegeneration^26,27^. Here, we attempted to address the following questions: (i) does 6-OHDA-triggered generation of ROS contribute to dysregulated autophagy? If yes, (ii) what is the potential role for ROS-induced dysregulated autophagy in the process of neuronal death? Using MN9D dopaminergic neuronal cells^28,29^, mouse embryonic fibroblast (MEFs) of *Atg5* knockout (KO) cells, and primary cultures of cortical neurons exposed to 6-OHDA, we found that ROS-dependent dysregulated autophagic flux contributed to capsase-3-dependent apoptosis. Intriguingly, this was quite contrary to our previous reports demonstrating that neuronal death caused by *N*-methyl-4-phenylpyridinium iodide (MPP^+^) was primarily accompanied by the impairment of autophagic flux, which was independent of caspase activation^30,31^.

## Results

### Autophagic events are associated with 6-OHDA-induced neurodegeneration

**Fig. 1:**
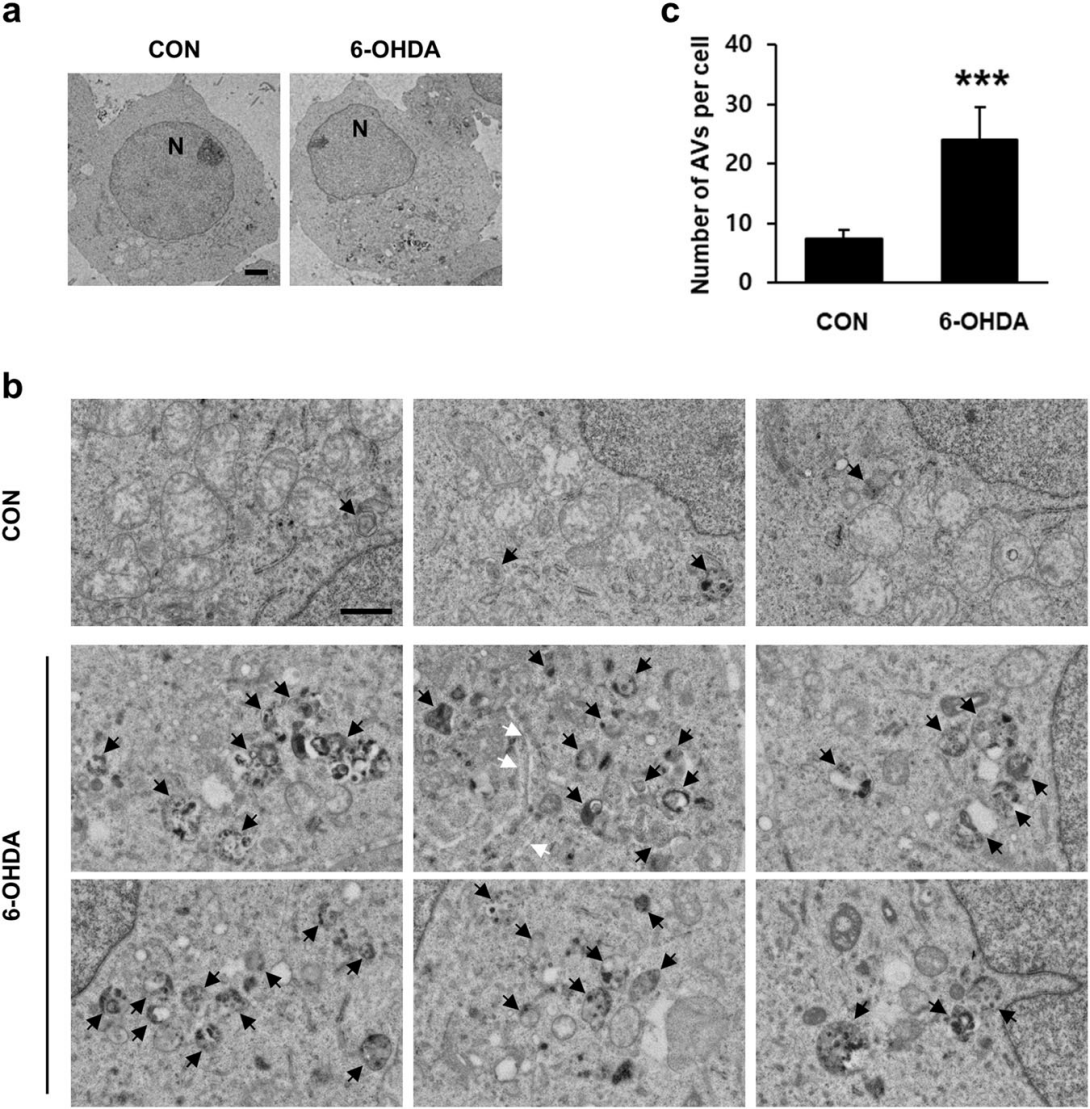
Ultrastructural features of autophagy induced by 6-OHDA in MN9D cells. **a** Electron micrographs were taken after treatment with or without 100 μM 6-OHDA for 15 h. N represents nucleus. Scale bar represents 2 μm. **b** Enlarged images of 6-OHDA-treated cells illustrate typical of autophagic vacuoles (AVs; black arrows) and phagophore (white arrows). Scale bar represents 1 μm. **c** The number of AVs was quantified from ten randomly selected cells per each group. Bars represent the mean ± standard deviation (7.4 ± 1.4 for untreated control vs. 24 ± 5.5 for 6-OHDA-treated cells). ***P *<* 0.001

To characterize 6-OHDA-induced autophagy in MN9D cells, we first examined ultrastructural changes associated with 6-OHDA-induced neurodegeneration. Using previously described approaches for ultrastructural characterization of autophagy^32–34^, we detected more autophagic vacuoles, including double-membraned autolysosomes, in MN9D cells treated with 6-OHDA compared to nontreated control cells (Fig. 1a). On closer inspection, we found that autophagic vacuoles were scattered around the cytosol (Fig. 1b; black arrows). Phagophores also were detected in the cytosol (Fig. 1b; white arrow). Enlarged electron microscopic images of autophagic vacuoles typically detected in 6-OHDA-treated cells were shown (Fig. S1). After analyzing ten randomly selected cells (ten serial ultrathin sections per cell), we found that the number of autophagic vacuoles was markedly increased after 6-OHDA treatment (Fig. 1c). In a separate study, immunoblot analyses were performed to measure the expression levels of Rab5 and Rab7 as markers of early endosome and late endosome/multivesicular bodies, respectively. No discernible changes in the expression levels of Rab5 and Rab7 were found in MN9D cells following 6-OHDA treatment (Fig. S2a). Immunocytochemistry also revealed that there were no obvious signs of colocalization of LC3 with either Rab5 or Rab7 in MN9D cells in either 6-OHDA-treated or 6-OHDA-nontreated cells (Fig. S2b, c).

**Fig. 2:**
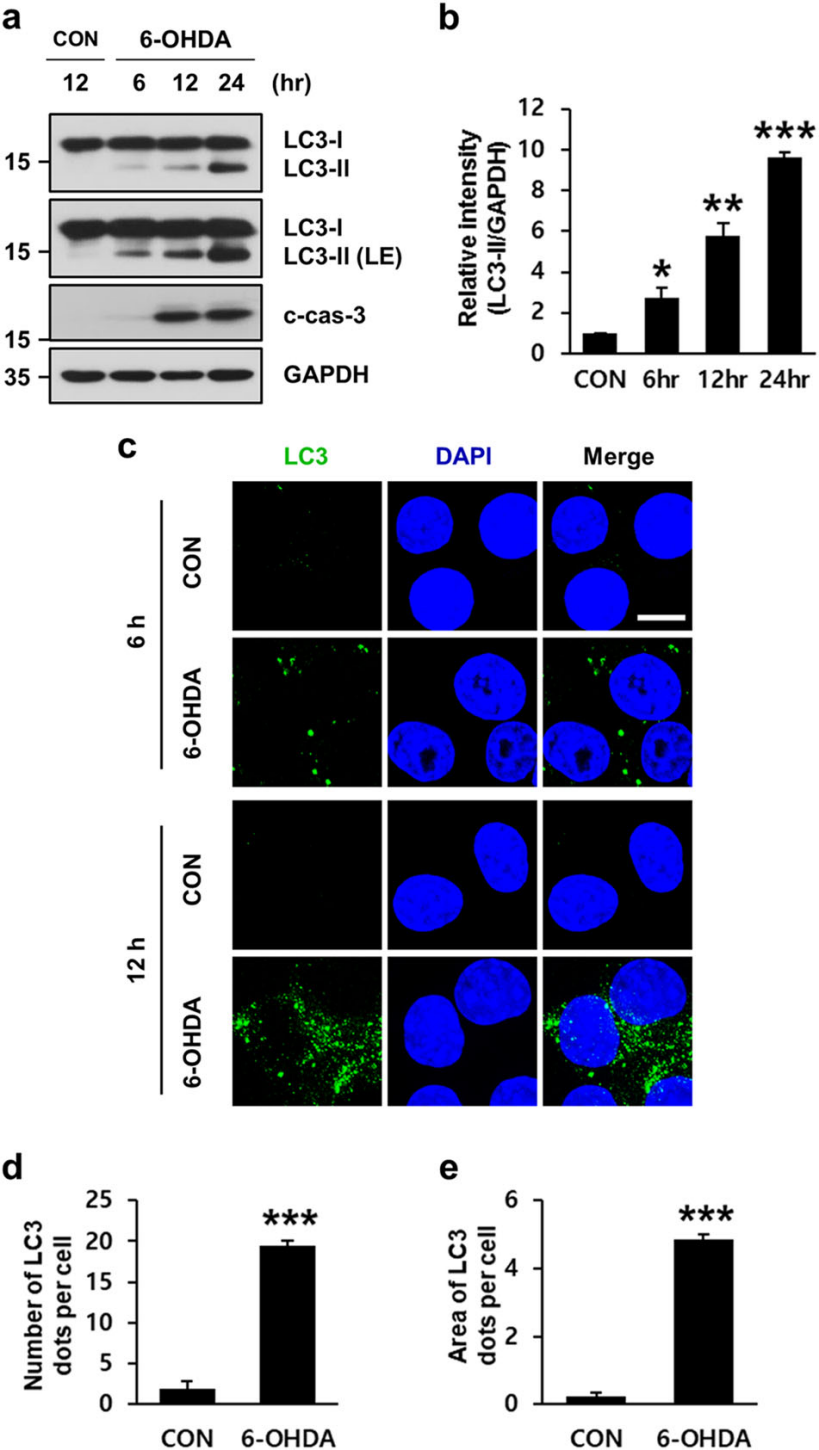
Biochemical characterization of autophagy induced by 6-OHDA in MN9D cells. **a** Cells were treated with or without 100 μM 6-OHDA for the indicated time periods. Immunoblot analyses were performed using anti-LC3 and anti-cleaved caspase-3 (c-cas-3) antibodies. Anti-GAPDH antibody was utilized as loading control. **b** The intensity of LC3-II signals at each timepoint was densitometrically measured using ImageJ, normalized by the intensity of GAPDH signal, and expressed as fold change relative to the untreated control value. Bars represent the mean ± standard deviation of three independent experiments (6 h, 2.7 ± 0.5; 12 h, 5.8 ± 0.6; 24 h, 9.7 ± 0.2). *P < 0.05; **P *<* 0.01; ***P *<* 0.001. **c** Cells treated with or without 100 μM 6-OHDA for the indicated time periods were subjected to immunocytochemical analyses using an anti-LC3 antibody (green) and nuclei counterstaining with Hoechst 33258 (blue). Cells were then examined under a confocal microscope. Merged images are provided in the right panels. Scale bars represent 10 μm. **d**, **e** The number (**d**) and area (**e**) of LC3 dots per cell were quantified using ImageJ in 100 μM 6-OHDA-treated cells for 12 h. Data are shown as the mean ± standard deviation of three independent experiments (number of LC3 dots, 2.0 ± 0.9 for control vs. 19.4 ± 0.7 for 6-OHDA-treated cell; area of LC3 dots, 0.2 ± 0.1 for control vs. 4.9 ± 0.1 for 6-OHDA-treated cell). ***P *<* 0.001

Microtubule-associated protein light chain 3 (MAP1-LC3/LC3) takes part in autophagosome biogenesis and substrate selection^32,33,35,36^. Cytoplasmic LC3-I is formed following Atg4-mediated C-terminal cleavage and conjugates with phosphatidylethanolamine to form lipidated LC3-II. LC3-II levels and LC3 puncta correlate with the number of autophagosomes. Therefore, we performed biochemical analyses of 6-OHDA-treated MN9D cells using an anti-LC3 antibody. LC3-II immunoreactivity appeared as early as 6 h after 6-OHDA treatment and progressively increased thereafter (Fig. 2a). Quantitative analyses showed that the relative levels of LC3-II increased during the incubation with 6-OHDA (Fig. 2b). This increase was parallel to the activation of caspase-3. Consistently, immunofluorescence analyses revealed that LC3 punctate staining was enhanced in 6-OHDA-treated MN9D cells compared to that in nontreated control cells (Fig. 2c–e). Taken together, our results indicated that morphological and biochemical changes typical of autophagy accompanied 6-OHDA-induced dopaminergic neurodegeneration.

### 6-OHDA-triggered ROS contributes to autophagic events that precedes caspase-dependent apoptosis

**Fig. 3:**
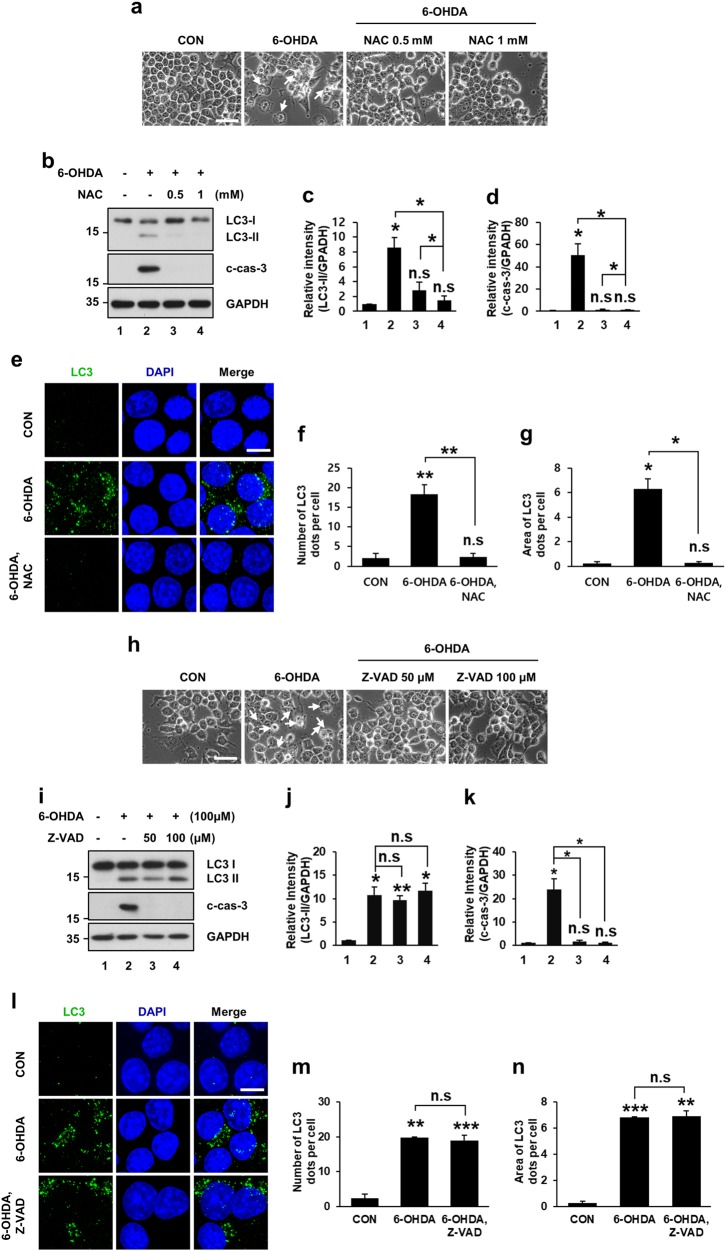
6-OHDA-triggered autophagy and cell death in MN9D cells is prevented by an antioxidant but not by a pan-caspase inhibitor. Cells were treated with 100 μM 6-OHDA for 12 h in the presence or absence of the indicated concentrations of the antioxidant *N*-acetyl-l-cysteine (NAC) (**a**−**g**) or the pan-caspase inhibitor Z-VAD-FMK (Z-VAD) (**h**−**n**). **a**, **h** Cells were then examined by a phase-contrast microscopy. Dying cells were indicated by white arrows. Scale bar represents 10 μm. **b**, **i** Immunoblot analyses were performed using anti-LC3 and anti-cleaved caspase-3 antibody (c-cas-3). **c**, **d**, **j**, **k** After normalization by GAPDH signal intensity, the relative intensity of LC3-II and c-cas-3 signals was expressed as a fold change relative to untreated control value. Bars represent the mean ± standard deviation of three independent experiments (LC3-ll, **c** 8.6 ± 1.4 for 6-OHDA-treated vs. 2.9 ± 1.0 for 6-OHDA plus 0.5 mM NAC-treated vs. 1.5 ± 0.5 for 6-OHDA-treated plus 1 mM NAC-treated cells; **j** 10.8 ± 1.7 for 6-OHDA-treated vs. 9.6 ± 0.97 for 6-OHDA plus 50 μM Z-VAD-FMK-treated vs. 11.7 ± 1.6 for 6-OHDA plus 100 μM Z-VAD-FMK-treated cells; c-cas-3, **d** 50.5 ± 10.2 for 6-OHDA-treated vs. 1.5 ± 0.5 for 6-OHDA plus 0.5 mM NAC-treated vs. 1.4 ± 0.1 for 6-OHDA plus 1 mM NAC-treated cells; **k** 24.0 ± 4.5 for 6-OHDA-treated vs. 1.6 ± 0.37 for 6-OHDA plus 50 μM Z-VAD-FMK-treated vs. 1.0 ± 0.3 for 6-OHDA plus 100 μM Z-VAD-FMK-treated cells). *P < 0.05; **P < 0.01; n.s. not significant. **e**, **l** After staining with an anti-LC3 antibody (green), confocal immunofluorescent images of LC3 were taken. Nuclei were counterstained with Hoechst 33258 (blue). Scale bar represents 10 μm. **f**, **g**, **m**, **n** The numbers of LC3 dots (**f**, **m**) and LC3 dot area (**g**, **n**) were then quantified using ImageJ. Bars represent the mean ± standard deviation of three independent experiments (number of LC3 dots, **f** 2.1 ± 1.2 for control vs. 18.4 ± 2.3 for 6-OHDA-treated vs. 2.4 ± 0.9 for 6-OHDA plus 1 mM NAC-treated cells; **m** 2.4 ± 1.1 for control vs. 19.8 ± 0.3 for 6-OHDA-treated vs. 19.0 ± 1.6 for 6-OHDA plus 100 μM Z-VAD-FMK-treated cells; area of LC3 dots; **g** 0.2 ± 0.1 for control vs. 6.3 ± 0.8 for 6-OHDA-treated vs. 0.3 ± 0.1 for 6-OHDA plus NAC-treated cells; **n** 0.3 ± 0.1 for control vs. 6.8 ± 0.1 for 6-OHDA-treated vs. 6.9 ± 0.4 for 6-OHDA plus Z-VAD-FMK-treated cells). *P < 0.05; **P *<* 0.01; ***P < 0.001; n.s. not significant

Previously, numerous studies demonstrated that ROS-mediated apoptotic signaling increased within several hours after 6-OHDA treatment^21,24,37,38^ and caused neuronal cell death^22–24,39,40^. Therefore, we investigated whether an antioxidant or a caspase inhibitor could affect 6-OHDA-induced autophagy. First, we treated MN9D cells with 6-OHDA in the presence or absence of *N*-acetyl-l-cysteine (NAC), a widely used antioxidant. As we previously demonstrated^22^, typical 6-OHDA-induced apoptotic morphological features such as shrinkage or condensation of the cytoplasmic membrane, were partially rescued following cotreatment with NAC (Fig. 3a). Interestingly, NAC cotreatment reduced not only drug-induced cleaved caspase-3 levels but also LC3-II levels (Fig. 3b–d). Similarly, immunocytochemical localization analyses revealed that 6-OHDA-induced increase in the number and area of LC3 dots was markedly reduced in the presence of NAC (Fig. 3e–g), indicating that ROS were responsible for autophagic events during 6-OHDA-induced cell death. We then used the pan-caspase inhibitor *N*-benzyloxycarbonyl-Val-Ala-Asp-fluoromethylketone (Z-VAD-FMK) to examine whether ROS-induced activation of caspase would affect 6-OHDA-induced autophagy. Cotreatment with Z-VAD-FMK prevented 6-OHDA-induced morphological changes and caspase-3 activation (Fig. 3h–k). However, Z-VAD-FMK had little effect on the LC3-II level, and number and area of LC3 dots (Fig. 3i–n), indicating that autophagic events preceded 6-OHDA-induced apoptosis.

### 6-OHDA treatment causes dysregulated autophagic flux

**Fig. 4:**
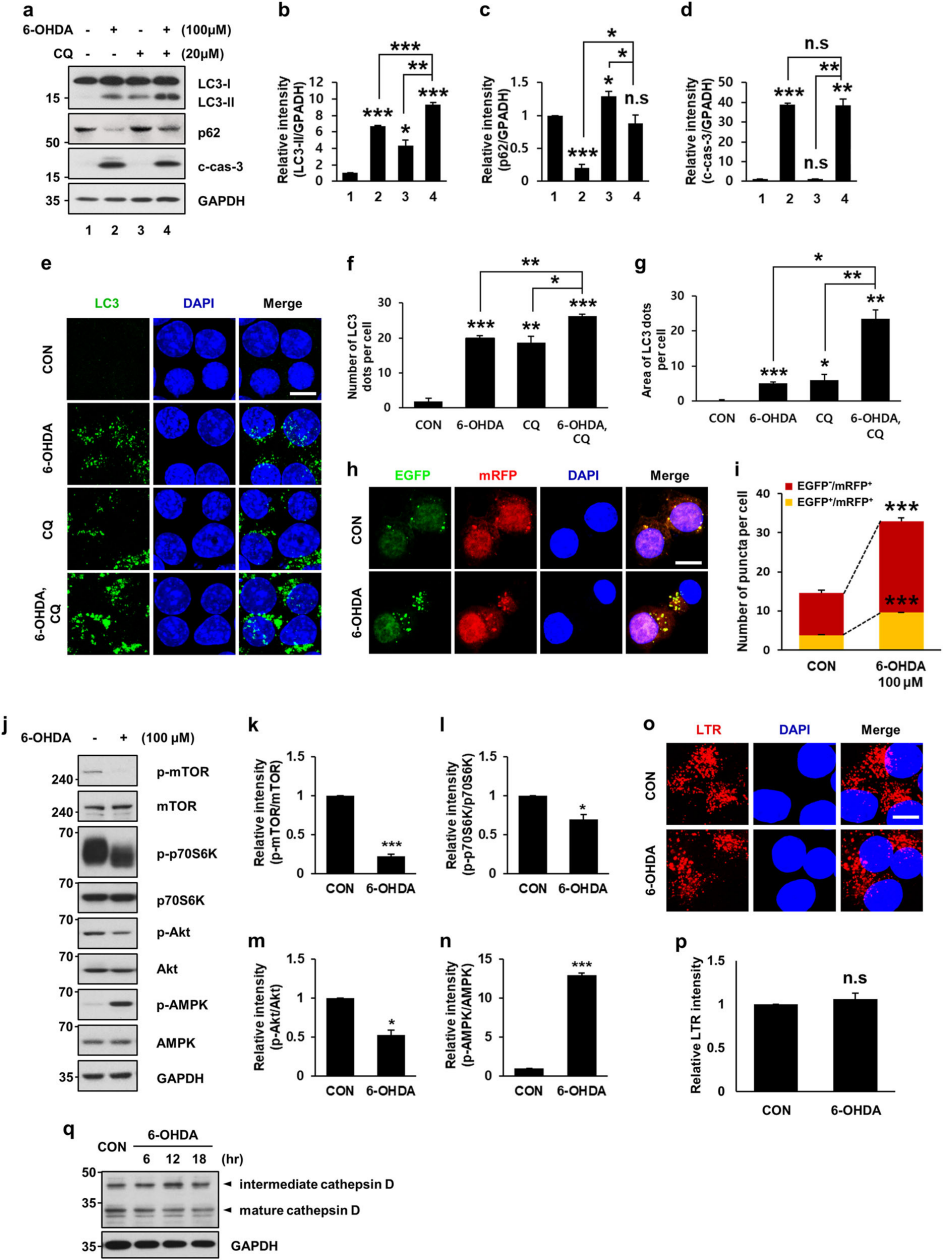
Dysregulated autophagy induced by 6-OHDA treatment. MN9D cells were incubated with 100 μM 6-OHDA for 12 h in the presence or absence of 20 μM chloroquine (CQ). **a** Autophagic flux in lysates was monitored and compared after immunoblotting with an anti-LC3 and anti-p62 antibody. Activation of caspase-3 was also measured using an anti-c-cas-3 antibody. **b**−**d** After normalization by GAPDH signal intensity, the relative intensity of LC3-II, p62, and c-cas-3 signals from each treatment was expressed as a fold change relative to untreated control value. Bars represent the mean ± standard deviation of three independent experiments (LC3-ll, 6.7 ± 0.1 for 6-OHDA-treated vs. 4.4 ± 0.6 for CQ-treated vs. 9.3 ± 0.2 for 6-OHDA plus CQ-treated cells; p62, 0.2 ± 0.1 for 6-OHDA-treated vs. 1.3 ± 0.1 for CQ-treated vs. 0.9 ± 0.1 for 6-OHDA plus CQ-treated cells; c-cas-3, 38.7 ± 1.0 for 6-OHDA-treated vs. 1.1 ± 0.1 for CQ-treated vs. 38.5 ± 3.1 for 6-OHDA plus CQ-treated group). *P < 0.05; **P < 0.01; ***P < 0.001; n.s. not significant. **e** Immunocytochemical localization of LC3 puncta was carried out following treatment with 100 μM 6-OHDA for 12 h in the presence or absence of CQ. Confocal immunofluorescence images represent LC3 dots (green) and nuclei (blue). Scale bar represents 10 μm. **f**, **g** The number (**f**) and area (**g**) of LC3 dots per cell were quantified using ImageJ. Bars represent the mean ± standard deviation of three independent experiments (number of LC3 dots, 1.9 ± 0.9 for control vs. 20.0 ± 0.8 for 6-OHDA-treated vs. 18.7 ± 1.8 for CQ-treated vs. 26.2 ± 0.5 for 6-OHDA plus CQ-treated cells; area of LC3 dots, 0.2 ± 0.1 for control vs. 5.1 ± 0.3 for 6-OHDA-treated vs. 6.0 ± 1.6 for CQ-treated vs. 23.4 ± 2.6 for 6-OHDA plus CQ-treated cells). *P < 0.05; **P < 0.01; ***P < 0.001. **h** MN9D cells were transiently transfected with the mRFP-EGFP-LC3 plasmid for 48 h and then treated with 100 μM 6-OHDA for 12 h. Confocal immunofluorescence images represent EGFP dots (green), mRFP dots (red), and nuclei (blue). Merged images are provided in the right panels. Scale bar represents 10 μm. **i** The number of puncta per cell that were EGFP^+^/mRFP^+^ or EGFP^-^/mRFP^+^ were quantified using ImageJ. Bars represent the mean ± standard deviation of three independent experiments (EGFP^+^/mRFP^+^, 3.8 ± 0.1 for control vs. 9.6 ± 0.1 for 6-OHDA-treated cells; EGFP^−^/mRFP^+^, 10.8 ± 0.7 for control vs. 23.4 ± 0.8 for 6-OHDA-treated cells). ***P < 0.001. **j** Following treatment with 100 μM 6-OHDA for 12 h, cellular lysates were blotted with antibodies against autophagy signaling molecules. **k**−**n** After normalization against intensity of total protein, the fold intensity of the phosphorylated forms was expressed as a fold change relative to untreated control value. Bars represent the mean ± standard deviation of three independent experiments (**k** 0.2 ± 0.03; **l** 0.7 ± 0.1; **m** 0.5 ± 0.1; **n** 13.0 ± 0.2 for 6-OHDA-treated cells). *P < 0.05; ***P *<* 0.001. **o** To assess lysosomal activity, MN9D cells exposed to 100 μM 6-OHDA for 12 h were stained with 0.5 μM LysoTracker® Red DND-99 (LTR) and subjected to confocal microscopy. Cell nuclei were counterstained with Hoechst 33258 (blue). Scale bar represents 10 μm. **p** LTR intensity was quantified from at least 100 randomly selected cells using ImageJ software, and expressed as the fold change relative to the untreated control value. Bar represents the mean ± standard deviation of three independent experiments (1.1 ± 1.3 for 6-OHDA-treated cells). n.s. not significant. **q** Following treatment with 100 μM 6-OHDA for the indicated time periods, expression of various forms of the lysosomal hydrolase cathepsin D was immunoprobed with an anti-cathepsin D antibody

Autophagic flux is a sequence of events in the autophagic pathway starting from the autophagosome formation to the degradation of autophagic substrates^41^. Accumulation of autophagosomes and increased levels of LC3-II indicate either activation of autophagic induction or blockade of downstream autophagic degradation steps^1,33^. To determine whether 6-OHDA-mediated accumulation of LC3-II and increase in LC3 dots are due to the activation of autophagy or blockade of lysosomal degradation, we monitored autophagic flux after cotreatment with the lysosomal inhibitor chloroquine (CQ)^32,42^. Immunoblot analyses indicated that LC3-II levels were higher in MN9D cells cotreated with 6-OHDA and CQ than in MN9D cells treated with 6-OHDA alone (Fig. 4a, b). The levels of p62/sequestosome-1 (p62), a well-known autophagic cargo protein^32^ were decreased in MN9D cells following 6-OHDA treatment and restored in CQ cotreated cells (Fig. 4a–c), indicating that p62 is rapidly degraded during 6-OHDA-induced autophagy. As demonstrated by others^15,16^, the restoration level of p62 in CQ cotreated cells was less than that in cells treated with CQ alone. No obvious difference in the levels of caspase activation was detected in cells treated with 6-OHDA alone or in combination with CQ (Fig. 4a–d). Unlike the previous report, which demonstrates caspase-dependent p62 cleavage^43^, no signs of p62 cleavage were detected in 6-OHDA-treated or 6-OHDA-nontreated cells (Fig. S3). Quantitative analyses demonstrated that the number and area of LC3 dots per cell were increased in MN9D cells cotreated with CQ compared to those in MN9D cells treated with 6-OHDA alone (Fig. 4e–g). To further support our hypothesis, we monitored autophagic flux using a tandem mRFP-EGFP-tagged LC3 probe^32,33^. Immunofluorescence and quantitative analyses of mRFP-EGFP-LC3 indicated that the number of autophagosomes (yellow dots, EGFP^+^/mRFP^+^) and autolysosomes (red dots, EGFP^−^/mRFP^+^) were increased following 6-OHDA treatment (Fig. 4h, i). Taken together, these data indicated that 6-OHDA treatment caused dysregulated autophagic flux in MN9D cells.

To obtain additional evidence of the dysregulated induction of autophagy in 6-OHDA-treated MN9D cells, we determined expression levels of the autophagic signaling molecules. Mammalian target of rapamycin (mTOR) is a master regulator of autophagy and phosphorylates p70S6K. Akt phosphorylates and thereby activates mTOR to inhibit autophagy, whereas AMP-activated protein kinase (AMPK), which is a key energy sensor and that regulates cellular metabolism to maintain energy homeostasis, promotes autophagy^44^. The activity of mTOR and Akt decreased following exposure to 6-OHDA as demonstrated by lower intensities of p-mTOR, p-p70S6K, and p-Akt bands, respectively (Fig. 4j–n). The levels of p-AMPK, the active form of AMPK, markedly increased following 6-OHDA treatment (Fig. 4j–n), supporting our hypothesis that 6-OHDA induced autophagic signaling. To determine whether the drug caused any changes in lysosomal activity, the cells treated with or without 6-OHDA were stained with LysoTracker Red. No significant difference was observed between fluorescent emissions of 6-OHDA-treated and control cells (Fig. 4o, p). The expression pattern of the lysosomal hydrolase cathepsin D after 6-OHDA treatment was similar to that observed in control cells (Fig. 4q), implying that lysosomal function remained relatively intact. Taken together, our results demonstrate that 6-OHDA-mediated accumulation of autophagosomes was caused by dysregulated de novo formation of autophagosomes. Intriguingly, this mechanism is quite contrary to the effect of other neurotoxins (such as MPP^+^ or nigericin), which led to LC3-II accumulation primarily due to impaired autophagic flux^30,31^.

### Inhibition of autophagy induction prevents 6-OHDA-induced apoptosis

**Fig. 5:**
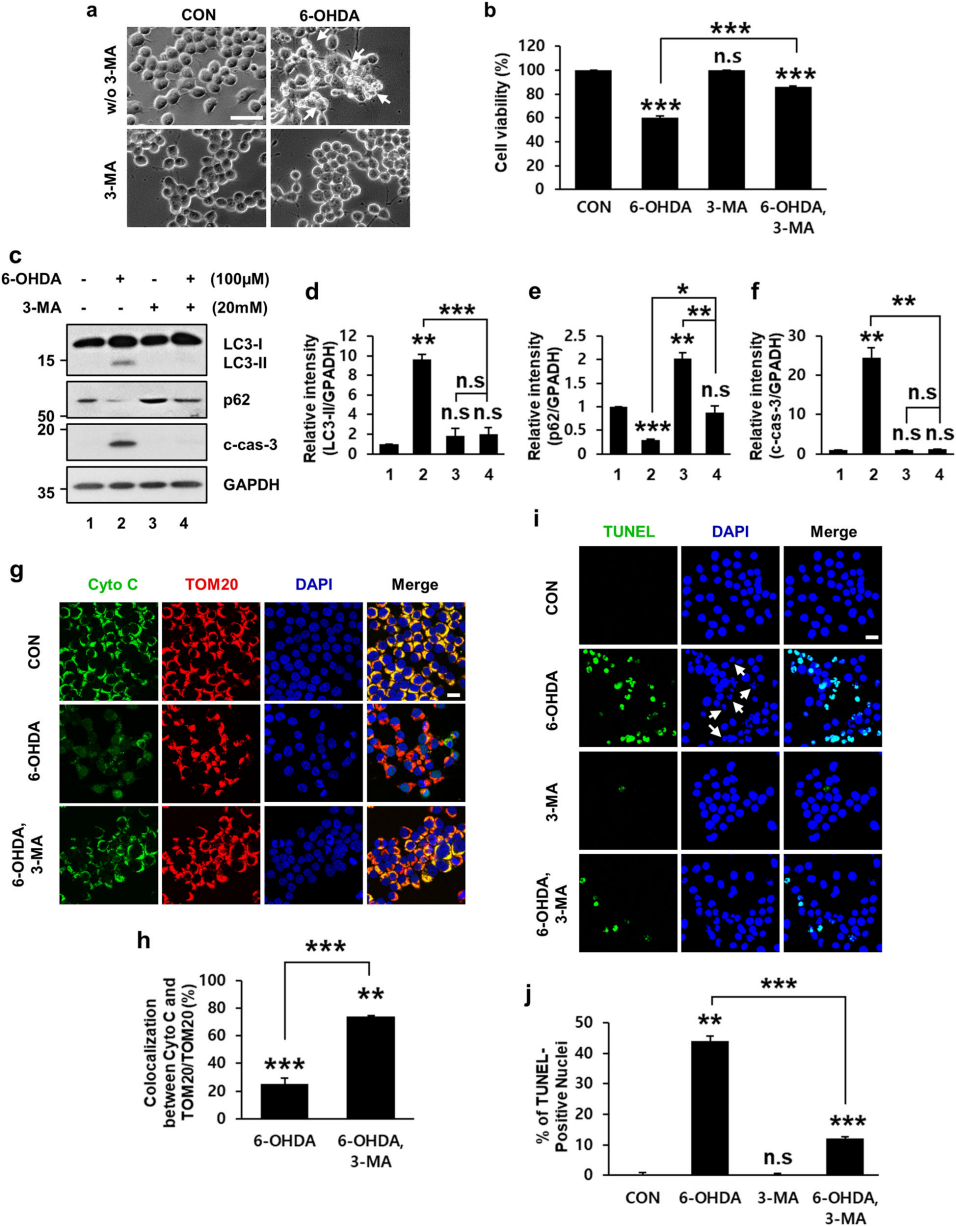
Rescue of 6-OHDA-induced neurodegeneration by 3-metyladenine (3-MA). MN9D cells were treated for 12 h with or without 100 μM 6-OHDA or 20 mM 3-MA alone or in combination. **a** Cells were analyzed by phase-contrast microscopy. Shrunken, phase-bright cells are indicated by white arrows. Scale bar represents 100 μm. **b** MTT reduction assays were performed to assess cell viability expressed as percentage over the untreated control cells (100%; 60.4 ± 1.3% for 6-OHDA-treated vs. 85.8 ± 0.8% for 6-OHDA plus 3-MA-treated cells). Bars represent the mean ± standard deviation of three independent experiments. ***P *<* 0.001; n.s. not significant. **c** Cell lysates were subjected to immunoblotting using anti-LC3, anti-p62 and anti-c-cas-3 antibodies. **d**−**f** After normalization against GAPDH, densitometric values of LC3-II, p62, and c-cas-3 signals were expressed as a fold change relative to untreated control value. Bars represent the mean ± standard deviation of three independent experiments (LC3-II, 9.6 ± 0.5 for 6-OHDA-treated vs. 1.9 ± 0.7 for 3-MA-treated vs. 2.0 ± 0.7 for 6-OHDA plus 3-MA-treated cells; p62, 0.3 ± 0.01 for 6-OHDA-treated vs. 2.0 ± 0.1 for 3-MA-treated vs. 0.9 ± 0.1 for 6-OHDA plus 3-MA-treated cells; c-cas-3, 24.6 ± 2.5 for 6-OHDA-treated vs. 0.9 ± 0.04 for 3-MA-treated vs. 1.1 ± 0.2 for 6-OHDA plus 3-MA-treated cells**)**. *P < 0.05; **P *<* 0.01; ***P *<* 0.001; n.s. not significant. **g**, **i** Confocal images of cytochrome *c* (cyto *c*, green), TOM20 (red) and TUNEL staining (green) in MN9D cells treated with 100 μM 6-OHDA alone or in combination with 20 mM 3-MA were shown. Nuclei were counterstained with Hoechst 33258 (blue). Merged images are shown in the right panel. White arrows indicate condensed or fragmented nuclei. Scale bar represents 20 μm. **h** The percentage of colocalization between cyto *c* and TOM20 over total TOM20 was expressed over the untreated control cells (100%). Confocal images of at least 30 randomly selected cells from each of the three independent experiments we used for quantitation. Bars represent the mean ± standard deviation of three independent experiments (25.4 ± 1.1% for 6-OHDA-treated vs. 74.1 ± 0.9% for 6-OHDA plus 3-MA-treated group). **P *<* 0.01; ***P *<* 0.001. **j** The percentage of TUNEL-positive nuclei was assessed from at least 100 randomly selected cells from each of the three independent experiments. Data were shown as the mean ± standard deviation of three independent experiments (0.4 ± 0.4% for untreated vs. 44.0 ± 1.7% for 6-OHDA-treated vs. 0.3 ± 0.2% for 3-MA-treated vs. 12.0 ± 0.6% for 6-OHDA plus 3-MA-treated cells). **P *<* 0.01; ***P *<* 0.001; n.s. not significant

To evaluate the functional role of dysregulated autophagic induction in 6-OHDA-triggered neuronal cell death, MN9D cells were incubated with 3-methyladenine (3-MA), a cell-permeable inhibitor of type III phosphatidylinositol 3-kinase (PI3K) that blocks autophagosome formation^45^. Blockade of autophagic induction by cotreatment with 3-MA prevented 6-OHDA-induced cell shrinkage, so that cotreated cells had morphological features quite identical to those of cells treated with 3-MA alone or nontreated control group (Fig. 5a). We then conducted the 3-(4,5-dimethylthiazol-2-yl)-2,5-diphenyltetrazolium bromide (MTT) reduction assays to determine whether inhibition by 3-MA of 6-OHDA-induced autophagic induction would affect cell death. We observed that MN9D cells cotreated with 3-MA showed increased resistance against 6-OHDA-induced toxicity (Fig. 5b).

To elucidate the mechanisms by which 3-MA prevented 6-OHDA-mediated cell death, we determined whether there was interplay between autophagy induction and caspase-3 activation. LC3-II levels in the group cotreated with 3-MA were markedly reduced (Fig. 5c, d). In contrast, 6-OHDA-induced decreases in p62 levels were partially recovered by cotreatment with 3-MA (Fig. 5c–e). Interestingly, 3-MA cotreatment also prevented 6-OHDA-triggered caspase-3 activation (Fig. 5c–f). We next inquired whether 3-MA cotreatment also blocked the release of cytochrome *c* to the cytosol, an event that triggers the onset of apoptosis by activating caspases^46–48^. Double immunofluorescence staining revealed that cytochrome *c* was colocalized with mitochondrial import receptor subunit TOM20 in untreated control cells (Fig. 5g, upper panel). Upon exposure to 6-OHDA, cytochrome *c* staining became diffused and not colocalized with TOM20 (Fig. 5g, middle panel), indicating that 6-OHDA treatment caused the release of cytochrome *c* to the cytosol. In contrast, cotreatment with 3-MA resulted in cytochrome *c* staining pattern quite similar to that observed in nontreated control cells (Fig. 5g, lower panels). More specifically, the quantification analyses revealed that the percentage of colocalization between cytochrome *c* and TOM20 over the total area of TOM20 was markedly decreased following 6-OHDA treatment but significantly restored by cotreatment with 3-MA (Fig. 5h). To further confirm whether 6-OHDA-induced dysregulated autophagic induction is linked to apoptotic cell death, TUNEL staining was conducted in MN9D cells treated with 6-OHDA in the presence or absence of 3-MA. The number of TUNEL-positive cells was dramatically increased after 6-OHDA treatment (Fig. 5i). Autophagic inhibition by cotreatment with 3-MA reduced the percentage of TUNEL-positive cells by approximately 30% (Fig. 5j). From these data, we hypothesized that 6-OHDA-induced dysregulated autophagic induction promoted caspase-3-dependent neuronal cell death.

**Fig. 6:**
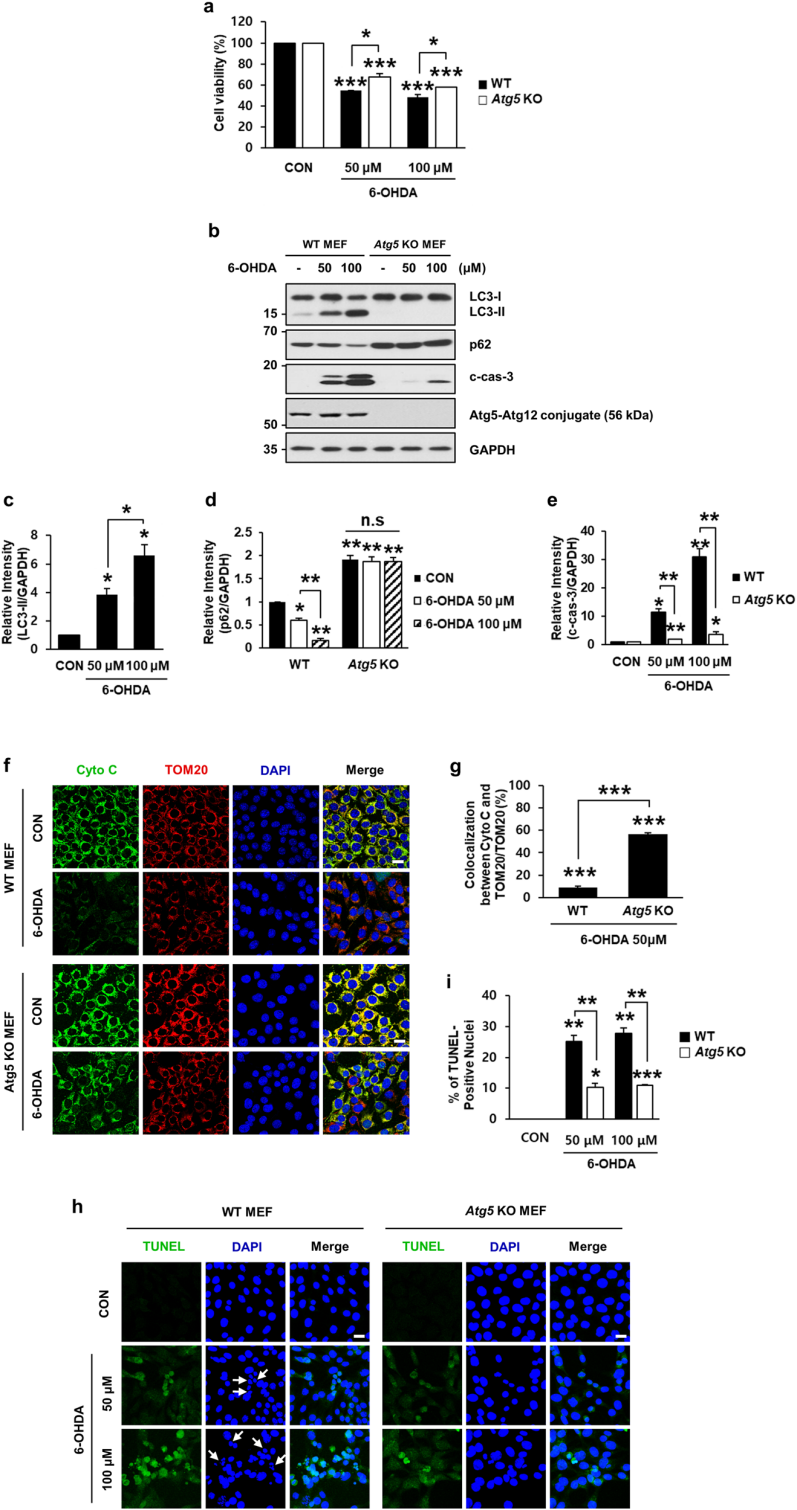
Rescue of 6-OHDA-induced neurodegeneration by knockout of *Atg5*. Wild-type (WT) and *Atg5* knockout (KO) MEFs were treated with 50 or 100 μM 6-OHDA for 12 h. **a** MTT reduction assays were performed to assess cell viability. Viability was expressed as percentage over each untreated control cell (100%). Bars represent the mean ± standard deviation of three independent experiments (at 50 μM 6-OHDA, 54.4 ± 0.4% for WT MEFs vs. 67.9 ± 2.9% for *Atg5* KO MEFs; at 100 μM, 6-OHDA 48.2 ± 2.5% for WT MEFs vs. 58.2 ± 0.1% for *Atg5* KO MEFs). *P < 0.05; ***P < 0.001. **b** Cell lysates were subjected to immunoblot analyses using anti-Atg5, anti-LC3, anti-p62, and anti-c-cas-3. **c**−**e** After normalization against GAPDH, densitometric values of LC3-II, p62, and c-cas-3 signals were expressed as a fold change relative to untreated control value. Bars represent the mean ± standard deviation of three independent experiments (LC3-II, 3.9 ± 0.4 for 50 μM 6-OHDA-treated vs. 6.6 ± 0.8 for 100 μM 6-OHDA-treated WT MEFs; p62, 0.6 ± 0.1 for 50 μM 6-OHDA-treated vs. 0.2 ± 0.04 for 100 μM 6-OHDA-treated WT MEFs vs. 2.0 ± 0.1 for untreated vs. 1.9 ± 0.1 for 50 μM 6-OHDA-treated vs. 1.9 ± 0.1 for 100 μM 6-OHDA-treated *Atg5* KO MEFs; c-cas-3, 9.8 ± 1.1 for 50 μM 6-OHDA-treated WT MEFs vs. 1.8 ± 0.1 for 50 μM 6-OHDA-treated *Atg5* KO MEFs; 29.4 ± 3.0 for 100 μM 6-OHDA-treated WT MEFs vs. 3.7 ± 0.8 for 100 μM 6-OHDA-treated *Atg5* KO MEFs**)**. *P < 0.05; **P *<* 0.01; n.s. not significant. **f** WT and *Atg5* KO MEFs exposed to 50 μM 6-OHDA for 12 h were subjected to immunofluorescent staining for cytochrome *c* (green), TOM20 (red), and nuclei (blue). Merged images are shown in the right panels. Scale bar represents 20 μm. **g** The percentage of colocalization between cyto *c* and TOM20 over total TOM20 was expressed over the untreated control cells (100%). Confocal images of at least 30 randomly selected cells from each of the three independent experiments were used for quantitation. Bars represent the mean ± standard deviation of three independent experiments (8.9 ± 0.8% for WT MEFs vs. 56.5 ± 0.6% for Atg5 KO MEFs). ***P < 0.001. **h** Confocal images of TUNEL assay in WT and *Atg5* KO MEFs exposed to 50 μM or 100 μM 6-OHDA for 18 h were examined for quantitation. White arrows indicate condensed or fragmented nuclei. Scale bar represents 20 μm. **i** The percentage of TUNEL-positive nuclei was assessed from at least 100 randomly selected cells from each of the three independent experiments. Data represent the mean ± standard deviation of three independent experiments (25.2 ± 2.0% for 50 μM 6-OHDA-treated WT MEFs vs. 10.3 ± 1.3 for 50 μM 6-OHDA-treated *Atg5* KO MEFs; 27.8 ± 1.6 for 100 μM, 6-OHDA-treated WT MEFs vs. 10.9 ± 0.4 for at 100 μM, 6-OHDA-treated *Atg5* KO MEFs). *P < 0.05; **P *<* 0.01; ***P < 0.001

Because pharmacological effects might be insufficiently specific, to strengthen out hypothesis, we used *Atg5* knockout (KO) MEFs in which autophagy cannot proceed due to the lack of Atg5 ^44^. First, we measured cell viability by the MTT reduction assay to determine whether 6-OHDA-induced cell death was prevented in *Atg5* KO MEFs. As shown in Fig. 6, we found that *Atg5* knockout itself did not affect cell viability. Compared with the viability of wild-type (WT) MEFs, deletion of *Atg5* partially protected against 6-OHDA-induced cell death (Fig. 6a). The phenotype rescue was even more prominent when expression levels of LC3-II, p62, and activated caspase-3 were compared in WT and *Atg5* KO MEFs. Immunoblot analyses revealed that 6-OHDA-induced accumulation of LC3-II, reduction in p62, and activation of caspase-3 were significantly blocked in *Atg5* KO MEFs (Fig. 6b–e). As determined by colocalization between cytochrome *c* and TOM20, we found that the 6-OHDA-induced release of cytochrome *c* to the cytosol in WT MEFs also was inhibited in *Atg5* KO MEFs (Fig. 6f, g). In support of these data, we found that the number of TUNEL-positive nuclei in WT MEFs following 6-OHDA treatment was significantly reduced in 6-OHDA-treated *Atg5* KO MEFs (Fig. 6h, i). Thus, both pharmacological and genetic experiments demonstrated that dysregulated autophagy induced by 6-OHDA contributed to caspase-3-dependent apoptosis.

### 6-OHDA-induced increase in autophagy promotes death of mouse primary cortical neurons

**Fig. 7:**
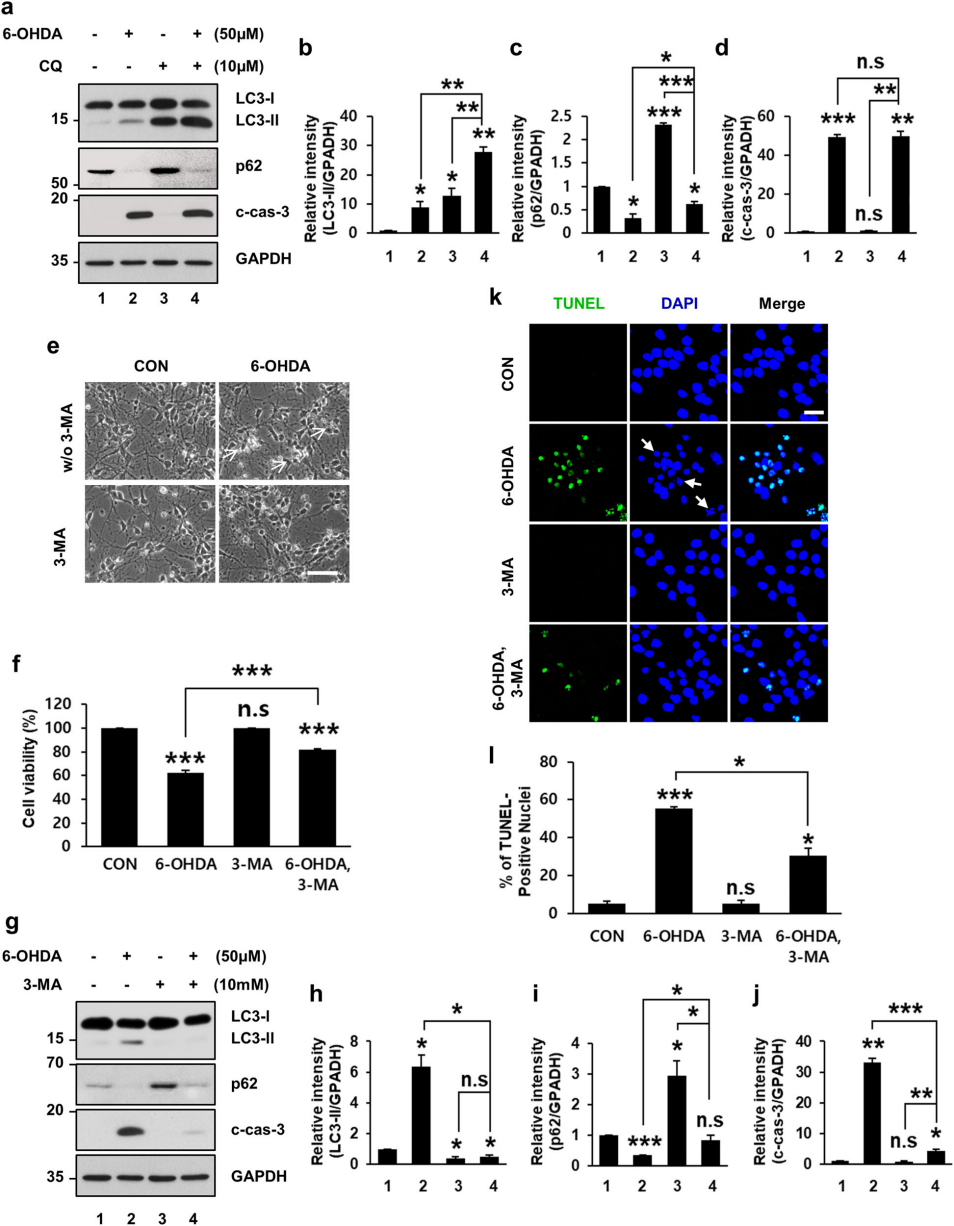
Rescue of 6-OHDA-induced cortical neuronal death by 3-MA. Primary cultures of mouse cortical neurons were incubated for 12 h with 50 μM 6-OHDA in the presence or absence of 10 μM CQ (**a**−**d**) or 10 mM 3-MA (**e**−**l**). **a** Cell lysates were subjected to immunoblotting using antibodies against LC3, p62, and c-cas-3. **b−d** After normalization against GAPDH signal intensity, the fold intensity of LC3-II, p62, and c-cas-3 signals were expressed as fold change relative to control value. Data represent the mean ± standard deviation of three independent experiments (LC3-II, 9.0 ± 1.8 for 6-OHDA-treated vs. 12.9 ± 2.4 for CQ-treated vs. 27.7 ± 1.7 for 6-OHDA plus CQ-treated cells; p62, 0.3 ± 0.1 for 6-OHDA-treated vs. 2.3 ± 0.03 for CQ-treated vs. 0.6 ± 0.1 for 6-OHDA plus CQ-treated group; c-cas-3, 49.5 ± 1.2 for 6-OHDA-treated vs. 1.2 ± 0.1 for CQ-treated vs. 49.6 ± 2.8 for 6-OHDA plus CQ-treated group). *P < 0.05; **P *<* 0.01; ***P *<* 0.001; n.s. not significant. **e** Mouse primary cortical neurons were analyzed by a phase-contrast microscopy after drug treatment. Shrunken, phase-bright cells are indicated by white arrows. Scale bar represents 50 μm. **f** Cell viability was measured by the MTT reduction assay. Viability was expressed as percentage over the untreated control cells (100%; 62.0 ± 2.2% for 6-OHDA-treated vs. 82.1 ± 0.3% for 6-OHDA plus 3-MA-treated group). Bars represent the mean ± standard deviation of three independent experiments. ***P *<* 0.001; n.s. not significant. **g** The levels of LC3-II, p62, and c-cas-3 were determined by immunoblotting. **h**−**j** The relative intensities of LC3-II, p62, and c-cas-3 signals were calculated after normalization against GAPDH signal intensity. Bars represent the mean ± standard deviation of three independent experiments (LC3-II, 6.3 ± 0.8 for 6-OHDA-treated vs. 0.4 ± 0.1 for 3-MA-treated vs. 0.5 ± 0.1 for 6-OHDA plus 3-MA-treated group; p62, 0.3 ± 0.001 for 6-OHDA-treated vs. 3.0 ± 0.5 for 3-MA-treated vs. 0.9 ± 0.1 for 6-OHDA plus 3-MA-treated group; for c-cas-3, 33.2 ± 1.3 for 6-OHDA-treated vs. 0.9 ± 0.2 for 3-MA-treated vs. 4.4 ± 0.5 for 6-OHDA plus 3-MA-treated group). *P < 0.05; **P < 0.01; ***P < 0.001; n.s. not significant. **k** TUNEL assay was performed in primary cultures of mouse cortical neurons exposed to 50 μM 6-OHDA for 18 h in the presence or absence of 10 mM 3-MA. White arrows indicate condensed or fragmented nuclei. Scale bar represents 20 μm. **l** The percentage of TUNEL-positive nuclei was assessed from at least 100 randomly selected cells from each of the three independent experiments. Data represent the mean ± standard deviation of three independent experiments (5.4 ± 1.3% for untreated vs. 55.6 ± 0.8% for 6-OHDA-treated vs. 5.2 ± 1.9% for 3-MA-treated vs. 30.5 ± 3.8% for 6-OHDA plus 3-MA-treated group). *P < 0.05; ***P < 0.001, n.s. not significant

Having established the sequence of events between dysregulated autophagic induction and apoptosis in MN9D cells and MEFs, we sought to translate these findings to primary cultures of cortical neurons. Following incubation with 6-OHDA, LC3-II levels increased and p62 levels decreased concomitantly with caspase-3 activation (Fig. 7a–d). Cotreatment of cortical neurons with CQ accelerated LC3-II accumulation and partially restored 6-OHDA-induced p62 degradation (Fig. 7a–c). No significant difference in caspase activation was detected in cortical neurons treated with 6-OHDA alone or in combination with CQ (Fig. 7a–d). These results indicated that autophagy induction after 6-OHDA treatment was also prominent in primary cultures of cortical neurons.

To investigate whether 6-OHDA-induced neuronal cell death was attributable to dysregulated autophagy, 3-MA was used to block autophagy in cortical neurons treated with 6-OHDA. A phase-contrast microscopy demonstrated that cell shrinkage caused by 6-OHDA was blocked in the presence of 3-MA (Fig. 7e). MTT reduction assays also revealed that 6-OHDA-mediated neuronal death was significantly blocked in the presence of 3-MA (Fig. 7f). We then analyzed whether neuronal protection by 3-MA was a consequence of inhibition of 6-OHDA-induced dysregulated autophagy and caspase-3 activation. Immunoblot analyses indicated that 6-OHDA-induced increase in LC3-II and cleaved caspase-3 was attenuated in the presence of 3-MA (Fig. 7g–j). In cortical neurons cotreated with 3-MA, 6-OHDA-induced decreases in p62 were restored to nontreated control levels (Fig. 7i). The TUNEL assay also indicated that 6-OHDA-induced DNA fragmentation was markedly inhibited in cortical neurons cotreated with 3-MA (Fig. 7k, l). In summary, our data provide evidence for a cytotoxic role of dysregulated autophagic induction in neuronal cells caused by the activation of downstream caspase-dependent pathway.

## Discussion

**Fig. 8:**
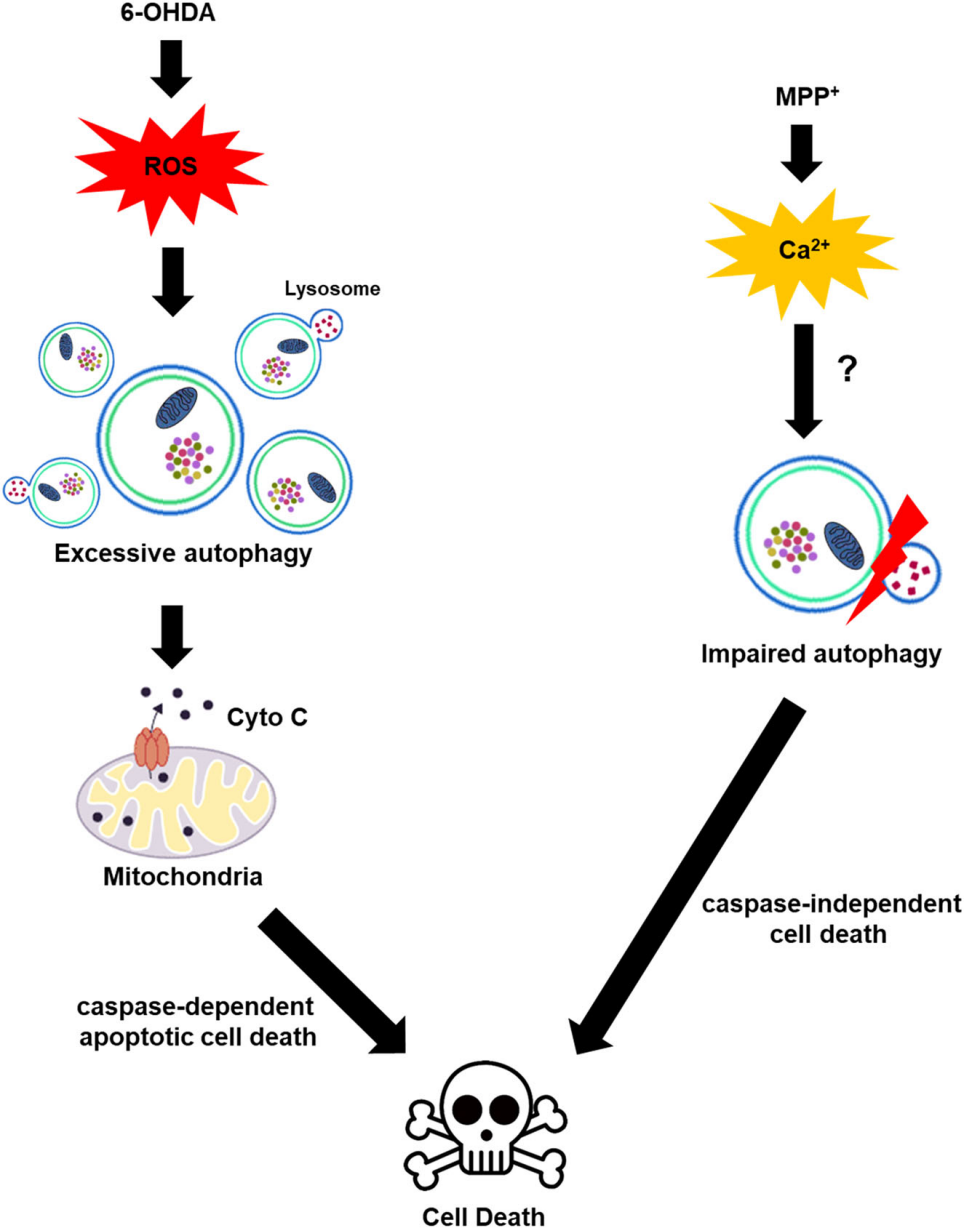
Schematic diagram of 6-OHDA-induced neurodegeneration. Previously, we demonstrated that 6-OHDA-induced generation of ROS triggered caspase-dependent neuronal death whereas MPP^+^-induced intracellular calcium surge caused caspase-independent cell death^22, 24, 26, 27, 60^. Based on the data obtained from pharmacological inhibition (3-MA, CQ) and genetic deletion (*Atg5*) experiments, in this study, we further propose that potentially damaging levels of ROS induced by 6-OHDA treatment trigger dysregulated autophagic induction and this upstream event, in turn, causes the release of cytochrome *c* from the mitochondria to the cytosol, eventually accelerating caspase-3-dependent apoptosis. This finding is in sharp contrast to our previous finding that MPP^+^ impaired autophagic flux by inhibiting lysosomal activity^30, 31^

Here, we demonstrated that exposure of MN9D cells, MEFs, and primary cortical neurons to 6-OHDA caused dysregulated autophagic induction and subsequent caspase-dependent apoptosis. Inhibition of autophagy by pharmacological and genetic means protected against 6-OHDA-induced cell death. Our present data are consistent with recent study by He et al.^13^, which also reported on the role of 6-OHDA-induced autophagy in neurotoxicity. The findings from this study and our study are in contrast to other reports^49^, which have shown that 6-OHDA induces autophagic flux dysfunction by impairing transcription factor EB activation and lysosomal function in dopaminergic neurons and SH-SY5Y cells. Although this discrepancy is not yet understood, our data—which was acquired from various cell types exposed to 6-OHDA—clearly demonstrate a temporal sequence of cell death that starts with autophagy induction and is followed by activation of apoptosis. Schweichel and Merker proposed that prenatal tissues undergo three morphologically distinct types of cell death in response to toxicants: type I (apoptotic cell death), type II (autophagic cell death (ACD)), and type III (necrotic cell death)^50^. Apoptosis is a highly regulated process that confers advantages during organism’s lifecycle. Indeed, apoptosis is considered as the dominant mechanism of neuronal homeostasis, the dysregulation of which is observed in neurodegenerative diseases including PD^51,52^. Numerous studies revealed that genetic mutations in PD-related genes led to apoptotic cell death^53–55^. Neurotoxin-induced models of PD suggested that apoptosis is primarily responsible for dopaminergic neurodegeneration^24,26,27^. Furthermore, it has been demonstrated that abnormal protein aggregation is commonly observed in various neurodegenerative disorders, and neurons can remove abnormal protein aggregates by two main degradation pathways: ubiquitin-proteasome system and autophagy^56,57^. In particular, autophagy plays an important role in the degradation of long-lived proteins and cellular components in postmitotic neurons^58^. Given the greater dependency of neurons on autophagy for their survival, abnormality in any autophagic flux step can lead to neurodegeneration^13,15,16,59^. In support of this notion, we have demonstrated that impairment of autophagic flux is responsible for dopaminergic neuronal cell death induced by neurotoxins, MPP^+^, and nigericin^30,31^. In those cases, we found that autophagic degradation process was usually impaired due to lysosomal dysfunction, which caused accumulation of LC3, p62, and ubiquitinated proteins. Intriguingly, that mechanism is in sharp contrast to the findings of the present study where 6-OHDA induced dysregulated autophagy in neurons but the lysosomal activity remained relatively intact. Because 6-OHDA and other neurotoxins with PD-like effects (e.g., MPP^+^, and rotenone) have been shown to cause cell death by distinct ways^22,24,26,27,60^, we hypothesize that ROS causes dysregulated autophagic induction, whereas intracellular calcium surge could be responsible for autophagic flux impairment (Fig. 8). It would be intriguing to investigate whether and how p62 dynamics are coupled with LC3 dynamics during parkinsonian mimetics-induced dysregulated autophagy.

ROS are produced as normal products of cellular metabolism and play important roles as signaling molecules regulating various biological phenomena^61^. Because of their highly reactive nature, ROS are directly or indirectly involved in promoting disease progression. In the substantia nigra pars compacta region of PD patients, high levels of ROS are detected, and numerous studies demonstrated that ROS indeed induce apoptosis^62^. Interestingly, recent studies have reported that ROS generation is linked to the induction of autophagic flux^63,64^. In the present study, we investigated the potential role of ROS in the regulation of autophagy using 6-OHDA-treated culture models of PD. We also attempted to reveal whether autophagic signaling contributes to neuronal apoptosis. As was shown by our ultrastructural and biochemical analyses, 6-OHDA-induced neurodegeneration was accompanied by increased autophagic flux, which, in turn depended on ROS generation. The results of our experiment in which 6-OHDA was cotreated with CQ showed that autophagosome accumulation was caused by the dysregulated induction of autophagy rather than by the impairment of autophagic flux. The findings of the study using a tandem mRFP-EGFP-tagged LC3 probe supported this hypothesis. Examination of both signaling molecules and lysosomal activity also supported our hypothesis that dysregulated autophagy was associated with 6-OHDA-induced neuronal death. Interestingly, we found that 6-OHDA-induced autophagy coincided with cytosolic release of cytochrome *c*, caspsase-3 activation, and DNA fragmentation in MN9D cells, MEFs, and primary cultures of cortical neurons. This indicated that autophagic events are somehow involved in 6-OHDA-induced apoptosis. By performing carefully designed experiments using inhibitors of either autophagy or apoptosis, we determined that dysregulated autophagic induction following 6-OHDA treatment was upstream of and linked to caspase-3-dependent apoptosis. Experiments with *Atg5* KO MEFs also indicated that autophagy induction preceded caspase-3-dependent apoptosis. Consequently, cotreatment with antioxidant blocked both autophagy induction and subsequent caspase-3 activation, indicating that ROS play a critical role in the regulation of autophagy and autophagy-mediated caspase-dependent neurodegeneration.

The concept of ACD has been initially formulated based on the observations of increased autophagy in dying cells^65^. However, some reports indicate that ACD is not sufficient to promote cell death independent of apoptosis or necrosis^66,67^. Even though the existence of ACD remains controversial, it is largely accepted that interplay between autophagy and apoptotic cell death pathway exists in the pathogenesis. Numerous studies have reported that autophagy precedes apoptosis and regulates apoptotic proteins^68,69^, or apoptosis regulates autophagy^70,71^. These proteins include Bcl-2 family, p53, death-associated protein kinase, and JNK^44^. Despite attempts to connect autophagy and apoptosis, their causal relationship wherein one process controls the other has not been adequately demonstrated. In our study, because neuronal death depended on 6-OHDA-induced caspase-3 activation, it is unclear whether dysregulated autophagy itself directly led to ACD. Nevertheless, we explicitly showed that dysregulated autophagy contributed to 6-OHDA-triggered caspase-3-dependent apoptosis. We confirmed this temporal cell death sequence by using pharmacological inhibition and genetic deletion experiments. For example, we demonstrated that cotreatment with 3-MA significantly blocked 6-OHDA-triggered caspase-3 activation and DNA fragmentation and partially protected cells against 6-OHDA toxicity in MN9D cells and mouse cortical neurons. We also found that 6-OHDA-induced caspase-3 activation, DNA fragmentation, and apoptotic neuronal death were attenuated when the induction of autophagy was prevented by the genetic depletion of *Atg5*. Moreover, we showed that the release of cytochrome *c* to the cytosol in response to 6-OHDA was significantly decreased in MN9D cells cotreated with 3-MA and in *Atg5* KO MEFs. Therefore, our results show a strong causal relationship between autophagy and apoptotic neuronal cell death in which ROS-triggered dysregulated autophagy promotes caspase-dependent apoptotic cell death (Fig. 8).

Further studies delineating the mechanisms underlying the potential role of dysregulated autophagy in apoptotic neurodegeneration are certainly required. First, it would be useful to determine whether autophagic vacuoles are potential sources of ROS^72,73^. It is possible that increased autophagic structures after the incubation with 6-OHDA set a time bomb for affected cells. Furthermore, it would be of immediate interest to reveal how ROS activates autophagy via recruiting protein kinases and downstream transcriptional factors^74,75^. Secondly, it has been proposed that many neurodegenerative diseases may result from dysregulated autophagy, particularly from the impairment of lysosomal degradative mechanisms^76,77^. Experimental evidence suggests that dysregulated autophagy puts a significant pressure on compromised lysosomes through increasing their cargos that needs to be degraded^73^. Therefore, it should be determined whether inhibition of dysregulated autophagy indeed provides benefit to cells by reducing the risk of lysosomal membrane destabilization^78^. Carefully designed experiments in these areas will expand our understanding of the role of autophagy in neurodegenerative disorders.

## Materials and methods

### Chemicals

The chemicals used for this study included 6-OHDA (Regis Chemical, Chicago, USA), chloroquine diphosphate salt (CQ; Sigma-Aldrich, St. Louis, MO, USA), NAC (Sigma-Aldrich), Z-VAD-FMK (Enzo Life Science Inc., Farmingdale, NY, USA), and 3-MA (Sigma-Aldrich). The concentrations and incubation periods of all drugs used in this study were empirically determined and used as described previously^22,24,26,27,30,31^.

### Cell culture and drug treatment

MN9D dopaminergic neuronal cell line was established by somatic fusion between embryonic mesencephalic neurons and N18TG neuroblastoma^28,29^ and cultured as previously described^30,31^. Briefly, MN9D cells were cultivated at 37 °C in Dulbecco’s modified Eagle’s medium (DMEM; Sigma-Aldrich) supplemented with 10% heat-inactivated fetal bovine serum (FBS; Gibco, Grand Island, NY, USA) on dishes coated with 25 µg/mL poly-d-lysine (Sigma-Aldrich) in the atmosphere of 90% air and 10% CO_2_. For WT and *Atg5* KO MEFs, the culture medium containing DMEM (GenDEPOT, Barker, TX, USA) supplemented with 10% FBS (GenDEPOT) was used, and the cells were incubated at 37 °C in the atmosphere of 95% air and 5% CO_2_. For drug treatment, culture medium was changed to N2 serum-free defined medium containing the indicated drugs and further incubated for the time periods indicated.

### Primary cultures of cortical neurons

All mice were handled in accordance with the guideline for animal care and use of the Yonsei University. All experimental procedures were approved by the Institutional Animal Care and Use Committee of the Yonsei University (permissions IACUC (2017-10-647-01 and 2018-01-689-01)). Cerebral cortices were removed from gestational day 14.5 mouse embryos (Orient, Gyeong-gi, Republic of Korea) and mechanically dissociated as previously described^79^. Briefly, dissociated cortical cells were plated at a density of 5×10^6^ cells per well of six-well plates or at 1×10^6^ cells per well of 24-well plates that were precoated with 100 μg/mL poly-d-lysine and 1 μg/mL laminin (Invitrogen, Carlsbad, CA, USA). Cultures were incubated at 37 °C in Minimum Essential Medium (MEM, Gibco) supplemented with 0.6% glucose (Sigma-Aldrich), 1 mM sodium pyruvate (Sigma-Aldrich), 2 mM l-glutamine (Sigma-Aldrich), penicillin-streptomycin (100 U/mL), and 10% FBS (Gibco) in the atmosphere of 95% air and 5% CO_2._ After 24 h, culture medium was changed to Neurobasal medium (Invitrogen) supplemented with 2% B-27 (Gibco), 0.5 mM l-glutamine and 10 μM cytosine β-d-arabinofuranoside (Ara-C, Sigma-Aldrich). At 4 DIV, cultures were treated with the indicated drugs that were dissolved in the same medium.

### Electron microscopy

For electron microscopy, MN9D cells grown in petri dishes were fixed with a mixture of 2% formaldehyde (freshly prepared from paraformaldehyde) and 0.2% glutaraldehyde in 0.1 M cacodylate buffer (pH 7.2) for 30 min at 37 °C. To stop fixation, free aldehyde groups were blocked by soaking the cells in 50 mM NH_4_Cl in cacodylate buffer for 1 h. After rinses with the buffer, the cells were mechanically removed, sedimented by centrifugation, enclosed in liquified 2% agarose, and postfixed with 1% osmium tetroxide (Electron Microscopy Science (EMS); Hatfield, PA, USA) in distilled water for 1 h, followed by en bloc staining for 1 h with 1% aqueous uranyl acetate (Heraeus, Hanau, Germany). After preparations were dehydrated in a series of graded ethanol, embedding in Epon-Araldite (Fluka, Buchs, Germany) was performed according to standard protocol. To unambiguously identify the autophagic structures, ten serial ultrathin sections (80 nm) were prepared on copper slot grids and stained with uranyl acetate and lead citrate and observed at 80 kV with a Hitachi H-7650 electron microscope (Hitachi, Tokyo, Japan). Ten randomly selected cells per each group were used for quantitation of autophagic vacuoles. Electron micrographs were taken with an 11 megapixel CCD XR611-M digital camera (Advanced Microscopy Techniques; Woburn, MA, USA).

### Phase-contrast and confocal microscopy

To observe morphological changes in MN9D cells and primary cultures of cortical neurons following treatment with the indicated drugs, the cells were photographed under an Axio Observer A1 microscope (Carl Zeiss, Zena, Germany). For immunocytochemical analysis, MN9D cells and MEFs were cultured on cover glasses coated with 100 μg/mL poly-d-lysine (Sigma-Aldrich). At time periods indicated, the cells were fixed in 4% paraformaldehyde (EMS) in PBS for 10 min at room temperature (RT). The permeabilization step was carried out with 0.1% saponin (Sigma-Aldrich) for 10 min at RT. Subsequently, the cells were blocked in PBS containing 0.2% Triton X-100 and 5% normal goat serum (Invitrogen), and then incubated with primary antibodies. These included a rabbit anti-LC3 antibody (Cell Signaling, Beverly, MA, USA; 1:200), a mouse anti-cytochrome *c* antibody (BD Biosciences, Bedford, MA, USA; 1:200), and a rabbit anti-TOM20 antibody (Santa Cruz Biotechnology, Inc., CA, USA; 1:200), in PBS containing 0.2% Triton X-100 and 1% normal goat serum at 4 °C for overnight. After extensive washes with PBS, the cells were incubated with appropriate secondary antibodies for 1 h at RT. These included Alexa 488-conjugated goat anti-rabbit IgG, Alexa 488-conjugated goat anti-mouse IgG, and Alexa 546-conjugated goat anti-rabbit IgG (all from Invitrogen, 1:200). Hoechst 33258 (Molecular Probes, Inc., Eugene, Oregon, USA; 1 μg/mL) was used for nuclei staining. After extensive washes with PBS, slides were mounted with Vectashield mounting medium (Vector Laboratories, Burlingame, CA, USA). Z stacked series of fluorescence images were acquired under a confocal microscope equipped with epifluorescence and digital image analyzer (LSM 700, Carl Zeiss). To quantify the LC3 punctate staining pattern per cell, at least 30 cells were randomly selected from each of three independent experiments and analyzed using ImageJ Imaging software (National Institutes of Health, Bethesda, MD, USA) as previously described^30^. Dots with diameters of 0.2–10 μm were included in our count. To assess the percentage of colocalization with cytochrome *c* and TOM20, at least 30 cells were randomly selected, and the areas of colocalized cytochrome *c* and TOM20 signal were divided by the total area of TOM20 signal using ImageJ software. MN9D cells treated with or without 6-OHDA were loaded with 0.5 μM LysoTracker Red DND-99 (LTR, Invitrogen), a fluorescent probe highly selective for acidic organelles, to conduct confocal imaging of lysosomal activity. The intensity of the LTR signals was quantified from at least 100 randomly selected cells from each of three independent experiments using ImageJ software. To explore the ectopic expression of a tandem mRFP-EGFP-LC3 probe, MN9D cells were transiently transfected using Lipofectamine^TM^ 2000 transfection reagent (Invitrogen). Following drug treatment, at least 30 randomly selected cells from each of three independent experiments were subjected to quantitative analyses for the number of puncta that were either EGFP^−^/mRFP^+^ or EGFP^+^/mRFP^+^.

### Immunoblot analysis

After drug treatment, the cells were washed with cold PBS (Lonza, Basel, Switzerland) with 2 mM ethylenediaminetetraacetic acid (EDTA; Sigma-Aldrich), lysed on ice in RIPA buffer (50 mM Tris-HCl [pH 7.4], 1% NP-40, 0.25% sodium deoxycholate, 150 mM NaCl, 1 mM EDTA, 0.1% sodium dodecyl sulfate (SDS)) containing complete protease inhibitor cocktail (Roche Applied Science, Mannheim, Germany), and then homogenized using a 1-mL syringe with a 20-gauge needle. Lysates were centrifuged at 13,000 × *g* for 20 min at 4 °C. Total supernatant protein was collected and quantified using Bradford protein assay reagent (Bio-Rad, Hercules, CA, USA). Predetermined amounts of protein from each preparation were separated on a sodium dodecyl sulfate polyacrylamide gel, transferred onto polyvinylidene fluoride membranes (Pall Corp., Ann Arbor, MI, USA), and blocked with Tris-buffered saline containing 0.1% Tween-20 (TBST) and 5% skim milk over 30 min. The membranes were incubated with primary antibodies overnight at 4 °C. Unless otherwise specified, all primary antibodies used were obtained from Cell Signaling. Primary antibodies used included a rabbit anti-LC3 antibody (1:4000), a rabbit anti-cleaved caspase-3 antibody (1:1000), a rabbit anti-p-mTOR antibody (1:1000), a rabbit anti-mTOR antibody (1:1000), a rabbit anti-p-p70S6K antibody (1:1000), a rabbit anti-p70S6K antibody (1:1000), a rabbit anti-p-AMPK antibody (1:1000), a rabbit anti-AMPK antibody (1:1000), a rabbit anti-p-Akt antibody (1:1000), a rabbit anti-Akt antibody (1:1000), a rabbit anti-Rab5 antibody (Cell Signaling, 1:1000), a rabbit anti-Rab7 antibody (Cell Signaling, 1:1000), a mouse anti-cathepsin D antibody (Santa Cruz; 1:1000), a guinea pig anti-p62/SQSTM1 (Progen, Heidelberg, Germany; 1:5000), a rabbit anti-Atg5 antibody (Novus Biologicals, Littleton, CO, USA; 1:1000) and anti-FLAG M2-Peroxidase (HRP) antibody (Sigma-Aldrich; 1:3000). Mouse anti-GAPDH (Millipore, Billerica, MA, 1:10,000) was used as loading control. After extensive washes with TBST, blots were incubated with horseradish peroxidase-conjugated anti-rabbit (Santa Cruz; 1:5000) or anti-mouse (Santa Cruz; 1:5000) secondary antibody. Specific bands were detected by using an enhanced chemiluminescence kit (ECL; PerkinElmer, Waltham, MA, USA). The relative band intensity was measured using ImageJ Imaging Software and expressed as a value normalized by the intensity of GAPDH signal.

### Viability assays

To examine cell viability after indicated drug treatments, MTT reduction assay and TUNEL assay were performed. For MTT reduction assay^80^, cells cultured on 24-well plates were incubated with 1 mg/mL MTT solution (Sigma-Aldrich) at 37 °C for 1 h and lysed for 18 h in an extraction buffer containing 20% SDS in 50% aqueous dimethylformamide. The optical densities of formazan grain were measured at 590 and 650 nm as test and reference wavelengths, respectively, by a VICTOR™ X5 Multilabel Plate Reader (PerkinElmer). Cell viability was expressed as percentage relative to the value in untreated control (100%). For TUNEL assays, cells were prepared using the same methods described previously for immunocytochemistry and confocal microscopy. After the permeabilization step, the cells were incubated with the In Situ Cell Death Detection Kit, Fluorescein (Roche) at 37 °C for 1 h. After extensive washes with PBS, the cells were incubated with PBS containing 1 μg/mL Hoechst 33258 (Molecular Probes, Inc.) at RT for 5 min, and then the slides were mounted with Vectashield mounting medium (Vector Laboratories). Z stacked series of fluorescence images were examined under a confocal microscope equipped with epifluorescence and digital image analyzer (LSM 700, Carl Zeiss). The percentage of TUNEL-positive nuclei was assessed from at least 100 randomly selected cells from each of three independent experiments.

### Statistics

Data are expressed as the mean ± standard deviation of three independent experiments. To determine the significance of differences between groups, the two-tailed Student’s *t*-tests or one-way analysis of variance followed by the Tukey’s post hoc test were performed by using Prism 6 (GraphPad Inc, San Diego, CA, USA). Statistical significance of differences was indicated as follows: ^***^P < 0.001; ^**^P < 0.01; or ^*^P < 0.05.

## Electronic supplementary material

Supplementary figures
