# Supplementary material for: Dysregulated autophagy contributes to caspase-dependent neuronal apoptosis
Source: Cell Death Dis. 2018 Dec 11;9(12):1189. doi: 10.1038/s41419-018-1229-y (PMC6289995; doi:10.1038/s41419-018-1229-y)
Supplement: Supplementary file 1 — Supplementary figures [file 41419_2018_1229_MOESM1_ESM.pptx]

## Slide 1
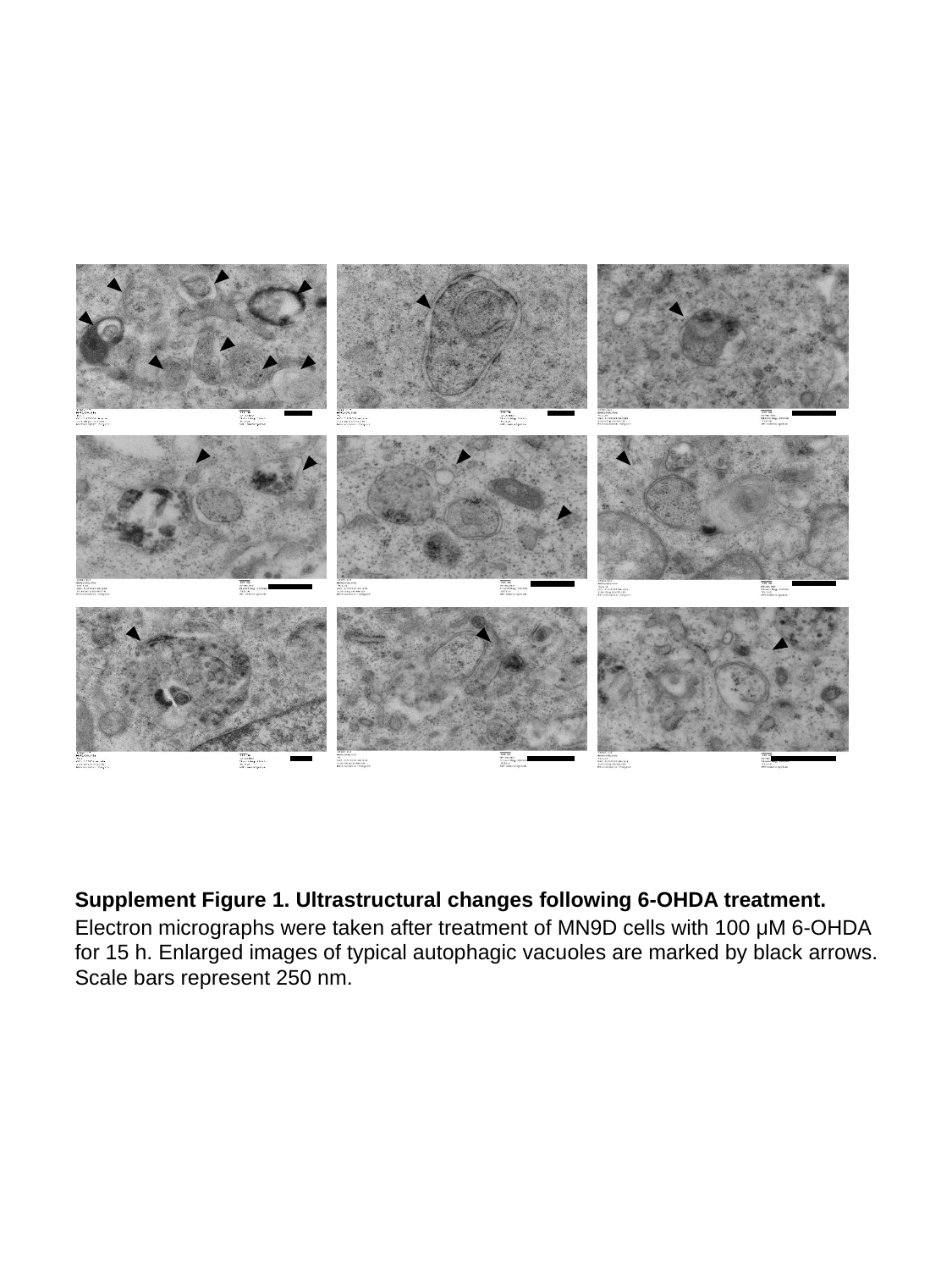

Supplement Figure 1. Ultrastructural changes following 6-OHDA treatment.
Electron micrographs were taken after treatment of MN9D cells with 100 μM 6-OHDA for 15 h. Enlarged images of typical autophagic vacuoles are marked by black arrows. Scale bars represent 250 nm.

## Slide 2
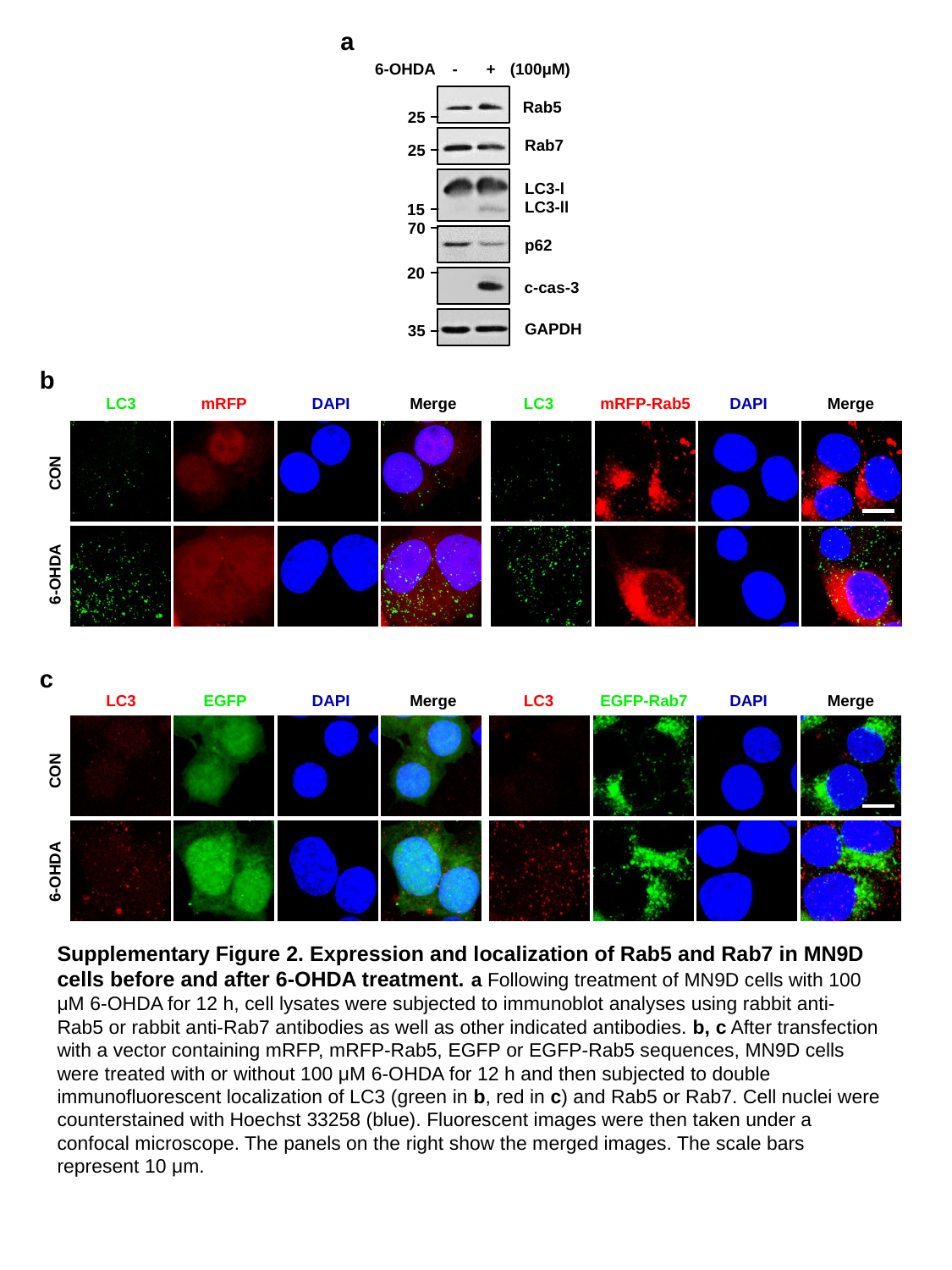

a
| 6-OHDA | - | + | (100μM) |
| --- | --- | --- | --- |
Rab5
25
Rab7
25
LC3-l
LC3-ll
15
70
p62
20
c-cas-3
GAPDH
35
b
LC3
mRFP
DAPI
Merge
LC3
mRFP-Rab5
DAPI
Merge
CON
6-OHDA
c
LC3
EGFP
DAPI
Merge
LC3
EGFP-Rab7
DAPI
Merge
CON
6-OHDA
Supplementary Figure 2. Expression and localization of Rab5 and Rab7 in MN9D cells before and after 6-OHDA treatment. a Following treatment of MN9D cells with 100 μM 6-OHDA for 12 h, cell lysates were subjected to immunoblot analyses using rabbit anti-Rab5 or rabbit anti-Rab7 antibodies as well as other indicated antibodies. b, c After transfection with a vector containing mRFP, mRFP-Rab5, EGFP or EGFP-Rab5 sequences, MN9D cells were treated with or without 100 μM 6-OHDA for 12 h and then subjected to double immunofluorescent localization of LC3 (green in b, red in c) and Rab5 or Rab7. Cell nuclei were counterstained with Hoechst 33258 (blue). Fluorescent images were then taken under a confocal microscope. The panels on the right show the merged images. The scale bars represent 10 μm.

## Slide 3
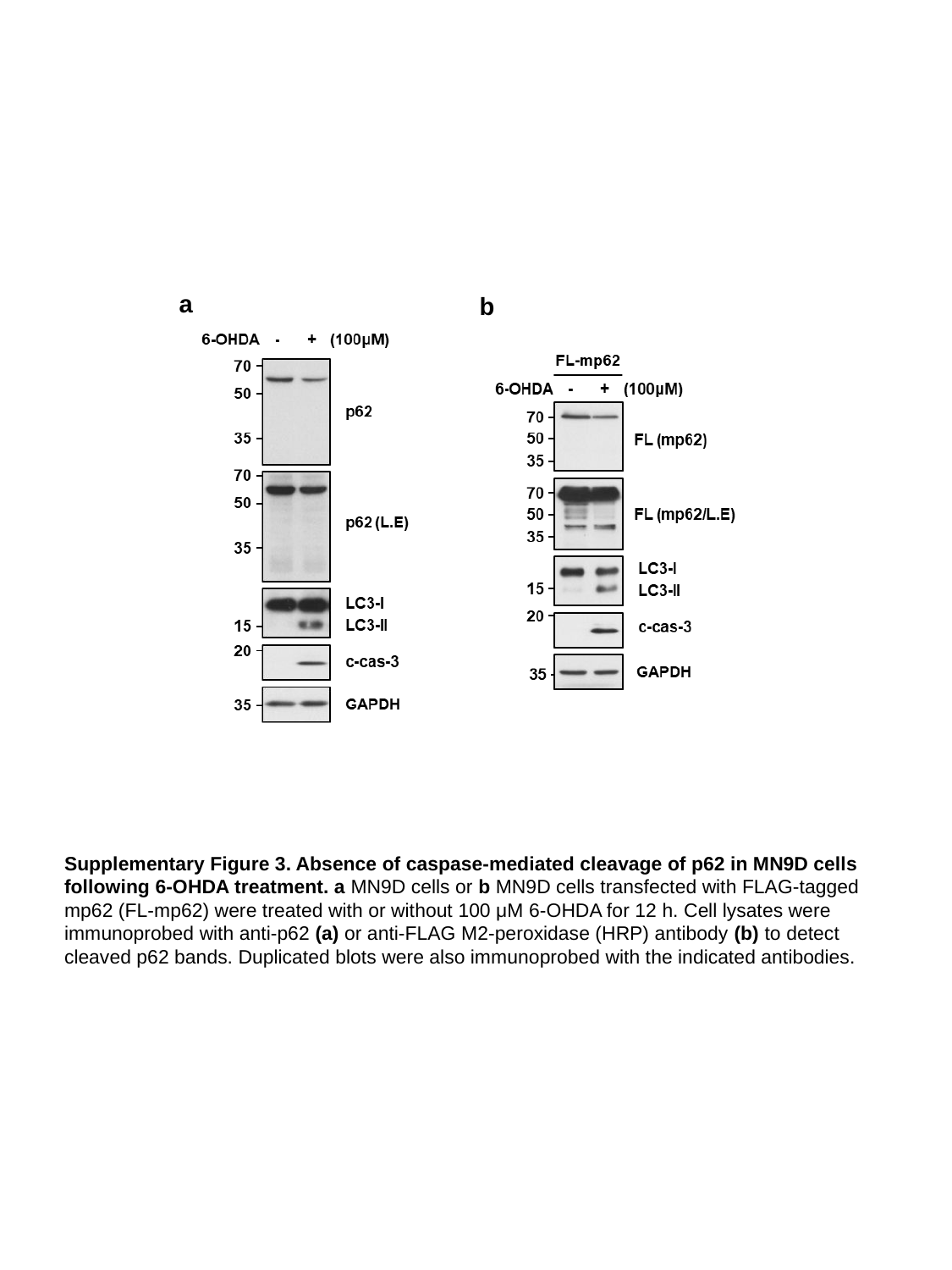

a
b
Supplementary Figure 3. Absence of caspase-mediated cleavage of p62 in MN9D cells following 6-OHDA treatment. a MN9D cells or b MN9D cells transfected with FLAG-tagged mp62 (FL-mp62) were treated with or without 100 μM 6-OHDA for 12 h. Cell lysates were immunoprobed with anti-p62 (a) or anti-FLAG M2-peroxidase (HRP) antibody (b) to detect cleaved p62 bands. Duplicated blots were also immunoprobed with the indicated antibodies.
